# Research on Tool Wear Prediction Method Based on CNN-ResNet-CBAM-BiGRU

**DOI:** 10.3390/s26020661

**Published:** 2026-01-19

**Authors:** Bo Sun, Hao Wang, Jian Zhang, Lixin Zhang, Xiangqin Wu

**Affiliations:** School of Mechanical and Vehicle Engineering, Changchun University, Changchun 130022, China; 230102024@mails.ccu.edu.cn (H.W.); 240101002@mails.ccu.edu.cn (J.Z.); z1456568492@163.com (L.Z.); 250102027@mails.ccu.edu.cn (X.W.)

**Keywords:** tool wear prediction, residual neural network, CBAM attention mechanism, bidirectional gated recurrent unit, hybrid deep learning

## Abstract

Aiming to address insufficient feature extraction, vanishing gradients, and low prediction accuracy in tool wear prediction, this paper proposes a hybrid deep neural network based on a Convolutional Neural Network (CNN), Residual Network (ResNet) residual connections, the Convolutional Block Attention Module (CBAM), and a Bidirectional Gated Recurrent Unit (BiGRU). First, a 34-dimensional multi-domain feature set covering the time domain, frequency domain, and time–frequency domain is constructed, and multi-sensor signals are standardized using z-score normalization. A CNN–BiGRU backbone is then established, where ResNet-style residual connections are introduced to alleviate training degradation and mitigate vanishing-gradient issues in deep networks. Meanwhile, CBAM is integrated into the feature extraction module to adaptively reweight informative features in both channel and spatial dimensions. In addition, a BiGRU layer is embedded for temporal modeling to capture bidirectional dependencies throughout the wear evolution process. Finally, a fully connected layer is used as a regressor to map high-dimensional representations to tool wear values. Experiments on the PHM2010 dataset demonstrate that the proposed hybrid architecture is more stable and achieves better predictive performance than several mainstream deep learning baselines. Systematic ablation studies further quantify the contribution of each component: compared with the baseline CNN model, the mean absolute error (MAE) is reduced by 47.5%, the root mean square error (RMSE) is reduced by 68.5%, and the coefficient of determination (R^2^) increases by 14.5%, enabling accurate tool wear prediction.

## 1. Introduction

With the shift toward intelligent and automated manufacturing, cutting tools remain fundamental to machining. Tool wear directly affects dimensional accuracy, surface integrity, and operational safety. Tool-related failures reportedly account for approximately 20% of machine-tool downtime. Although tooling costs represent only 3–5% of total manufacturing costs, their indirect impact on production efficiency can reach 15–20% [1,2]. These factors motivate the development of high-accuracy methods for tool wear monitoring and prediction.

Traditional tool condition monitoring approaches can be broadly divided into direct measurement and indirect measurement. With advances in sensing technologies and data-driven modeling, deep learning-based tool wear prediction has become an active research area.

For single-architecture networks, Cao et al. [3] designed a one-dimensional CNN to directly process vibration signals after wavelet transformation, avoiding manual feature design and achieving a classification accuracy of 98.28% in milling experiments. However, a plain CNN is often insufficient to capture temporal dependencies. Zhang et al. [4] proposed a tool condition monitoring method combining wireless sensor networks with deep learning, improving prediction accuracy via multi-scale feature extraction; nevertheless, temporal information was still not effectively exploited. Zhou et al. [5] employed a Long Short-Term Memory (LSTM) network to model the temporal evolution of tool wear under variable working conditions, where long-term dependencies can be captured through memory mechanisms. However, unidirectional LSTMs cannot leverage future context, and deeper recurrent networks may suffer from vanishing gradients during training. Li et al. [6] recently proposed a deep convolutional network that made progress in tool wear monitoring, but the overall architecture remains relatively simple.

Regarding hybrid architectures, a number of methods have emerged in recent years. Wang et al. [7] proposed a deep heterogeneous Gated Recurrent Unit (GRU) model, in which statistical features were first extracted via feature engineering and then temporal relationships were modeled using GRU. This approach performed well in complex-part milling but lacked an effective mechanism for adaptive feature reweighting. Caggiano et al. [8] developed an integrated tool wear monitoring approach for CFRP/CFRP stack drilling by combining autoencoders for automatic feature extraction with a memory-based GRU network for wear estimation, using multi-sensor signals such as force, torque, vibration, and acoustic emission. Their results highlight the effectiveness of representation learning and temporal modeling for tool wear prediction. Nevertheless, the approach is largely driven by latent representation learning for drilling signals and does not explicitly incorporate an attention mechanism to adaptively reweight informative features across modalities. Xu et al. [9] designed an intelligent monitoring model integrating multi-sensor features and enhancing informative features through deep learning and feature fusion; however, it primarily emphasized spatial representations and was relatively weak in temporal modeling. Meanwhile, hybrid models combining Transformers and CNN-Transformers have recently been applied to tool wear prediction to better capture long-term temporal dependencies and cross-sensor interactions. A representative hybrid design is CTNN (Liu et al., 2021) [10], which combines a Transformer branch (with positional encoding and multi-head self-attention) and a CNN branch in parallel, followed by feature fusion for regression. Notably, CTNN performs explicit temporal feature extraction and dimensionality reduction prior to learning by segmenting raw signals and computing statistical descriptors to obtain truncated sequences. This approach mitigates overfitting issues with excessively long raw signals but introduces additional preprocessing dependencies. Unlike hybrid CNN-Transformer models that primarily rely on self-attention for global sequence modeling, this approach employs CBAM as a lightweight feature reweighting mechanism on convolutional feature maps and utilizes BiGRU to model bidirectional temporal dependencies. This aims to achieve a superior balance between accuracy and efficiency to meet practical industrial deployment requirements. Chen et al. [11] explored the use of Deep Belief Networks in multi-sensor data fusion, but their residual-connection design was relatively simple and did not incorporate spatial attention. Zhao et al. [12] recently proposed a multi-scale spatio-temporal feature fusion network, achieving notable progress by combining parallel convolution with bidirectional recurrent networks; nevertheless, the architecture is relatively complex and training efficiency remains a concern.

Recent advances suggest that attention mechanisms play an increasingly important role in tool wear prediction. Guo et al. [13] introduced self-attention into tool condition monitoring, enhancing the model’s ability to focus on wear-related information. Zhou et al. [14] proposed a multi-head attention convolutional network and reported strong performance in 2023. In terms of residual optimization, Zhao et al. [15] improved the connection strategy of deep residual networks, offering insights into stabilizing deep model training. Liang et al. [16] proposed an adaptive ResNet that performed well in industrial big-data analysis.

Meanwhile, new hybrid paradigms continue to emerge. Li et al. [17] combined graph neural networks with convolutional networks to explore spatial correlations in tool wear. Gao et al. [18] proposed a spatio-temporal co-attention mechanism in 2024, providing a new approach for multi-sensor fusion. For bidirectional temporal modeling, De Barrena et al. [19] improved the structure of bidirectional GRU, enhancing long-term dependency modeling. Huan et al. [20] recently proposed a lightweight hybrid architecture that improves inference speed while maintaining accuracy. In feature engineering, Wang et al. [21] developed an adaptive multi-domain feature selection algorithm, and Sun et al. [22] proposed an intelligent feature extraction framework for high-dimensional feature processing.

Based on the above review, we observe that existing deep learning-based tool wear prediction methods—especially hybrid architectures—still face three key challenges:

(1) Limited depth of feature extraction: Many models employ shallow or relatively simple feature extractors. When the network is deepened, vanishing gradients or degradation may occur, restricting the extraction of discriminative representations from complex multi-domain signals [5,6]. (2) Lack of adaptive screening for key features: Many approaches do not include effective attention mechanisms to emphasize wear-relevant information and suppress redundant features. For example, Wang et al. [7] lacks feature reweighting, and Xu et al. [9] does not achieve collaborative optimization across multiple attention dimensions. (3) Insufficient synergy between feature learning and temporal modeling: Many hybrid designs are simple concatenations of modules rather than a tightly coupled end-to-end pipeline from deep feature extraction and adaptive feature screening to bidirectional temporal dependency modeling, which limits representational power and prediction accuracy [7,12].

To address these issues, this paper proposes a hybrid tool wear prediction method based on CNN–ResNet–CBAM–BiGRU, integrating: (i) a CNN-based feature extractor, (ii) ResNet-style residual connections for stable deep feature learning, (iii) CBAM for channel and spatial attention, and (iv) a BiGRU for bidirectional temporal modeling. The main contributions are as follows:

(1) A four-component hybrid architecture that jointly leverages convolution, residual learning, attention mechanisms, and bidirectional recurrent modeling for tool wear prediction;
(2) A 34-dimensional multi-domain feature system covering time-domain, frequency-domain, and time–frequency-domain characteristics to comprehensively describe wear-related patterns;
(3) ResNet-style residual connections to alleviate vanishing gradients and enable deeper feature extraction;
(4) CBAM-based dual attention to adaptively enhance informative features and suppress redundancy;
(5) BiGRU-based bidirectional temporal modeling to capture both forward and backward dependencies and improve prediction accuracy.

The organizational structure of this paper is as follows: Section 2 presents the theoretical background; Section 3 describes the network architecture and prediction workflow of the tool wear prediction method; Section 4 conducts experimental validation using a publicly available tool dataset to demonstrate the feasibility and effectiveness of the proposed model; Section 5 presents the conclusions of the proposed method.

## 2. Theoretical Basis

### 2.1. Theory of Multi-Domain Feature Extraction

Multi-domain feature extraction is widely used in signal processing to characterize signals from complementary perspectives, including statistical properties, spectral distribution, and time–frequency localization [23]. In tool wear monitoring, multi-sensor signals collected during cutting contain rich wear-related information distributed across different domains [24]. Features extracted from a single domain are often insufficient: time-domain descriptors mainly reflect amplitude statistics, frequency-domain descriptors lose time localization, and transient events are better represented in the time–frequency domain. Therefore, jointly describing multiple domains is beneficial for modeling the complex and non-stationary wear process [25,26].

In this study, a three-domain feature extraction framework is established to generate a 34-dimensional feature vector for each sensor channel. Seven sensor signals are used: cutting forces (Fx, Fy, Fz), vibration accelerations (Ax, Ay, Az), and acoustic emission RMS (AE_rms). For each signal, a fixed-length segment of *n* = 10,000 samples is used (from the 50,000th to the 60,000th samples in each record), and the sampling frequency is *f*_*s* = 50 kHz. The signal segment is denoted as *s* = [*s*_1, *s*_2, …, *s*_*n*].

Time-domain features (18): Twelve time-domain statistical features are extracted, including mean absolute value, maximum, minimum, root mean square (RMS), root amplitude, skewness-related statistic, kurtosis-related statistic, variance, median, range, zero-crossing count, and signal energy. In addition, six waveform factor features—shape factor, impulse (pulse) factor, skewness factor, crest factor, clearance factor, and kurtosis factor—are computed to further quantify waveform characteristics.

Frequency-domain features (8): The signal is transformed into the frequency domain using the Fast Fourier Transform (FFT). Based on the spectrum, eight frequency-domain descriptors are calculated to reflect the distribution of frequency components, including center frequency, mean-square frequency, root-mean-square frequency, frequency variance, spectral centroid, spectral bandwidth, spectral flatness, and peak frequency.

Time–frequency features (8): To capture non-stationary behaviors, a three-level wavelet packet decomposition is performed using the Daubechies 3 (db3) wavelet, resulting in eight sub-bands. An 8-dimensional sub-band feature vector is constructed using the energy-related magnitude of each sub-band.

Finally, a feature-level fusion strategy is adopted by concatenating the 34-dimensional feature vectors from the seven sensor channels, yielding a 238-dimensional fused representation (7 × 34) for each sample.

### 2.2. ResNet Residual Network

**Figure 1:**
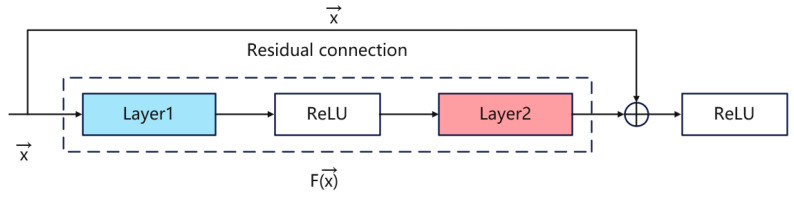
Residual structure diagram.

CNNs have strong feature extraction capabilities. To improve their performance, it is often necessary to increase the depth of the network. However, as the depth increases, the training error also continues to grow, thus making the network prone to problems such as gradient explosion or gradient vanishing [26]. Even with the initialization of the original data and regularization operations, the problem of network degradation still occurs. Residual networks add skip connections on the basis of linear network structures, thereby establishing an identity mapping learning relationship, making the input and output approximately the same. This method of feature fusion through addition can increase the depth of the network while improving its performance. The residual block structure used in this paper is shown in Figure 1 and adopts an improved residual learning strategy. The input data first passes through a 3 × 3 convolutional layer with a stride of 2 for downsampling, and then successively passes through two residual learning units. Each residual learning unit contains a 3 × 3 convolutional layer, a batch normalization (BN) layer, and a ReLU activation function. While the main branch is learning features, the skip connection directly transmits the input features to the output end, achieving feature fusion through addition, and finally passes through a ReLU activation function to obtain the final output [27]. This design of the residual block fully utilizes the advantages of skip connections, not only effectively alleviating the problem of gradient vanishing in the training of deep networks, but also making the network easier to optimize through the residual learning mechanism. The design of the stride convolution in it takes into account the needs of feature extraction and dimension adjustment, ensuring that the network can maintain good feature expression ability during the deepening process.

Figure 1 also illustrates the input–output flow of the residual block. Each block contains a main path and a skip connection: the main path performs nonlinear mapping via two 1 × 3 convolutions and two BN layers, representing the residual function. ReLU is used as the activation function. The skip connection adds the input to the learned residual; when channel dimensions differ, a 1 × 1 convolution is applied to align channels and enable valid element-wise addition.

The residual structure can be expressed as

(1) $$y=F\left(x,w_{i}\right)+x$$

During backpropagation, suppose the loss function is *K*, the gradient of the loss function with respect to the input can be expressed as

(2) $$\frac{\partial K}{\partial x}=\frac{\partial K}{\partial y}\frac{\partial y}{\partial x}=\frac{\partial K}{\partial y}\left(1+\frac{\partial F\left(x,w_{i}\right)}{\partial x}\right)$$

Equation (2) can be interpreted as two terms: the skip-connection term enables gradients to be propagated directly to shallow layers regardless of the main-path weights, thereby avoiding gradient loss. The second term depends on the main-path parameters and is not guaranteed to equal −1, so gradients through the main path may still attenuate when weights become small. Consequently, the skip connection provides a stable gradient route that mitigates multiplicative attenuation in backpropagation and helps stabilize training of deep networks.

### 2.3. CBAM Attention Mechanism

**Figure 2:**
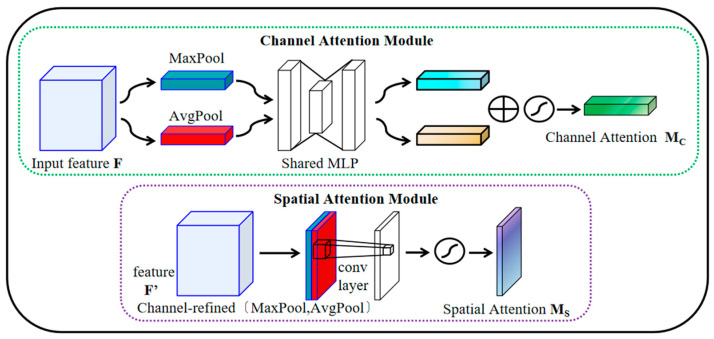
CBAM Attention Module Structure Diagram.

CBAM is a lightweight feature refinement module that combines channel attention and spatial attention, originally proposed by Woo et al. in 2018 [28,29]. As shown in Figure 2, it consists of two submodules: the channel attention module (CAM) and the spatial attention module (SAM) [30,31]. Given an intermediate feature map, CAM learns channel-wise importance to produce a channel attention map, whereas SAM highlights informative spatial locations to produce a spatial attention map. Applying attention in both dimensions allows CBAM to emphasize regions of interest and improve feature representation [32]. In practice, the input feature map is first reweighted by CAM and then refined by SAM through element-wise multiplication, making the feature processing more adaptive. CAM exploits inter-channel correlations to emphasize task-relevant channels and strengthen discriminative responses [33]. Its formulation is given in Equation (3): global average pooling and global max pooling are followed by a shared multi-layer perceptron (MLP), whose outputs are summed and passed through a sigmoid function to obtain the channel attention map.

(3) $$\begin{array}{cc} M_{c}(F) & =\sigma \{\mathrm{MLP}[\mathrm{AvgPool}(F)]+\mathrm{MLP}\times [\mathrm{MaxPool}(F)]\}\\ & =\sigma \left\{W_{1}\left[W_{0}\left(F_{\mathrm{avg}}^{c}\right)\right]+W_{1}\left[W_{0}\left(F_{\max}^{c}\right)\right]\right\} \end{array}$$

In Equation (3), $M_{c}$ denotes the channel attention map, and σ(⋅) is the sigmoid function. $F_{avg}^{c}$ and $F_{max}^{c}$ represent the channel descriptors obtained by global average pooling (AvgPool) and global max pooling (MaxPool), respectively, and MLP denotes the shared multi-layer perceptron. SAM further learns a spatial attention map by adaptively weighting spatial locations, thereby enhancing regions that are more relevant to the current task.

(4) $$M_{s}\left(F_{1}\right)=\sigma \left(f^{7\times 7}\left\{\mathrm{AvgPool}\left(F_{1}\right);\mathrm{MaxPool}\left(F_{1}\right)\right\}\right)=\sigma \left\{f^{7\times 7}\left(\left[F_{1\mathrm{avg}}^{s};F_{1\max}^{s}\right]\right)\right\}$$

In Equation (4): $F_{1\mathrm{avg}}^{s}\in {\mathbb{R}}^{1\times H\times W}$ and $F_{1\max}^{s}\in {\mathbb{R}}^{1\times H\times W}$ are the 2D feature maps generated by average pooling and max pooling along the channel dimension, respectively. $f^{7\times 7}$ denotes a convolution operation with a 7 × 7 kernel used to produce the spatial attention map. CBAM is a lightweight plug-and-play module that can be integrated into CNN-based feature extractors to refine intermediate feature maps via attention.

### 2.4. Bidirectional Gated Recurrent Unit (BiGRU)

**Figure 3:**
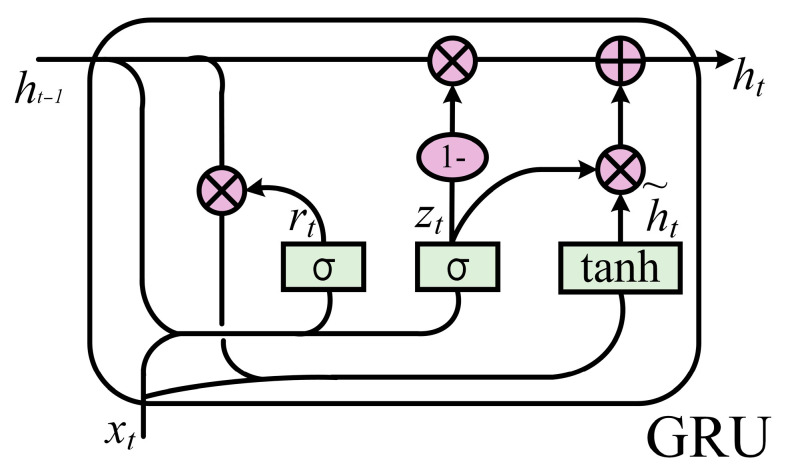
GRU Network Architecture Diagram.

The bidirectional gated recurrent unit (BiGRU) extends the GRU by processing a sequence in both forward and backward directions, which helps alleviate vanishing gradients in long sequences. A GRU cell uses two gates—the update gate and the reset gate—to control information retention and forgetting over time. Figure 3 shows the GRU structure, and the corresponding equations are provided below.

(5) $$\left\{\begin{array}{l} z_{t}=\sigma \left(w_{z}\cdot [h_{t-1},x_{t}]\right)\\ r_{t}=\sigma \left(w_{r}\cdot [h_{t-1},x_{t}]\right)\\ {\hat{h}}_{t}=\tanh\left(w\cdot [r_{t}⊙h_{t-1},x_{t}]\right)\\ h_{t}=(1-z_{t})h_{t-1}+z_{t}⊙{\hat{h}}_{t} \end{array}\right.$$

In Equation (5), $z_{t}$ and $r_{t}$ denote the update and reset gates, respectively; σ(⋅) is the sigmoid function and tanh(⋅) is the hyperbolic tangent function; W and U are learnable parameter matrices; $x_{t}$ is the input; and ${\tilde{h}}_{t}$ is the candidate hidden state at time t.

Based on the standard GRU, BiGRU processes the sequence in both forward and backward directions and fuses the two hidden states, enabling more comprehensive modeling of temporal correlations in the input sequence [34]. This makes BiGRU suitable for capturing long-term dependencies and bidirectional patterns in time series.

**Figure 4:**
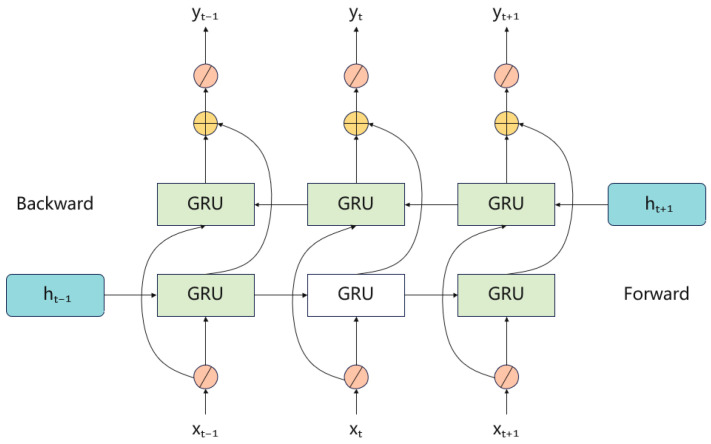
Bidirectional gated recurrent network structure diagram.

In the bidirectional structure, ${\vec{h}}_{t}$ and ${\overset{\leftarrow }{h}}_{t}$ represent the hidden states from the forward and backward passes, respectively. The BiGRU output at time t is obtained by combining ${\vec{h}}_{t}$ and ${\overset{\leftarrow }{h}}_{t}$ (e.g., via a weighted sum), as shown in Equations (6)–(8). Figure 4 illustrates the unit structure of BiGRU.

(6) $${\vec{h}}_{t}=\mathrm{GRU}\left(x_{t},{\vec{h}}_{t-1}\right)$$

(7) $${\overset{\leftarrow }{h}}_{t}=\mathrm{GRU}\left(x_{t},{\overset{\leftarrow }{h}}_{t-1}\right)$$

(8) $$h_{t}=w_{t}{\vec{h}}_{t}+v_{t}{\overset{\leftarrow }{h}}_{t}+b_{t}$$

By combining bidirectional information flow with gating mechanisms, BiGRU can selectively retain salient information and model long-term dependencies in time-series data.

## 3. The Design of CNN-ResNet-CBAM-BiGRU Model

This study proposes a CNN–ResNet–CBAM–BiGRU architecture that integrates convolution, residual learning, attention, and bidirectional recurrent modeling for tool wear prediction. The design targets three common limitations when processing multi-sensor features: insufficient feature extraction, weak modeling of long-term dependencies, and limited ability to emphasize wear-sensitive patterns. By combining these complementary modules, the network achieves accurate and stable tool wear prediction. Specifically, a residual CNN extracts spatial features, CBAM reweights important responses, and a BiGRU captures temporal dependencies, forming an end-to-end mapping from multi-sensor features to tool wear values.

Deep learning provides powerful nonlinear function approximation for complex input–output relationships. Its key idea is to learn hierarchical feature representations through multi-layer neural networks, enabling end-to-end mapping from inputs to outputs [25]. Here, deep learning serves as the core modeling approach for tool wear monitoring. Compared with traditional pipelines, it reduces reliance on manual feature engineering and can improve generalization and prediction accuracy.

To further improve performance, this study incorporates ResNet, CBAM, and BiGRU into a unified framework. ResNet mitigates vanishing gradients via residual connections, CBAM enhances salient features through dual attention, and BiGRU captures long-term dependencies by bidirectional processing. Based on these components, we develop a CNN–ResNet–CBAM–BiGRU tool wear monitoring model that leverages the synergy among modules for high-precision prediction. The model is evaluated on a public dataset to verify its effectiveness.

**Figure 5:**
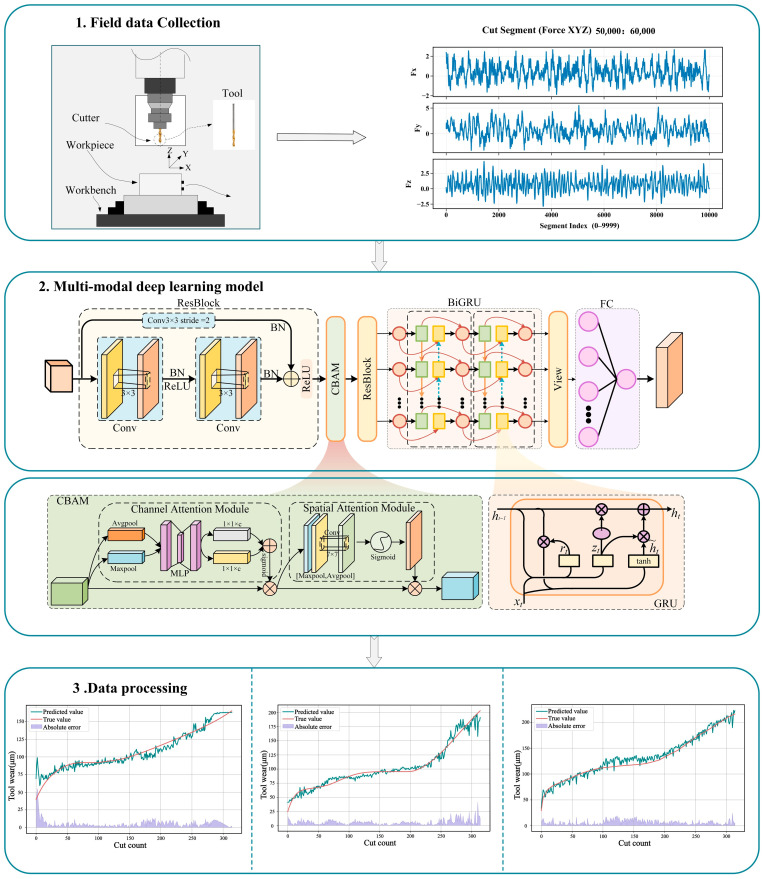
Overall model architecture diagram.

The proposed CNN–ResNet–CBAM–BiGRU model adopts an end-to-end learning strategy to automatically extract informative representations from multi-sensor inputs and learn a nonlinear mapping to tool wear values. The overall workflow is shown in Figure 5.

Step 1: Import the multi-sensor dataset and preprocess the signals/features. Input features are standardized using z-score normalization to place different dimensions on a comparable scale and to reduce the effect of magnitude differences during training.

Step 2: Format the preprocessed data for CNN input. The original three-dimensional feature tensor is expanded to a four-dimensional tensor so that subsequent convolution operations can be applied consistently.

Step 3: Feed the formatted tensor to the residual convolutional feature extractor. A two-layer ResNet is used for progressive feature extraction. The first stage expands channels from 1 to 16 to enrich representations, and the second stage reduces channels back to 1 to match the input requirements of the following modules.

Step 4: Pass the ResNet features to the CBAM. CBAM contains channel attention and spatial attention, applied sequentially to provide dual attention refinement. Channel attention first selects informative feature channels, and spatial attention then highlights important spatial positions.

Step 5: Reshape the attention-enhanced features into a temporal form suitable for recurrent modeling. Specifically, the sensor-channel dimension is treated as the time step, and the feature dimension is used as the input vector at each step.

Step 6: Input the temporal features to a bidirectional GRU for sequence modeling. A two-layer BiGRU simultaneously exploits forward and backward temporal information, improving long-term dependency modeling.

Step 7: Use the hidden state at the final time step as the global feature representation. This representation aggregates information from the entire sequence and is used for the final regression.

Step 8: Map the high-dimensional representation to a one-dimensional wear value using a fully connected layer. A single-layer regressor is adopted to limit parameter growth and reduce the risk of overfitting.

Step 9: Output the final wear prediction. The output is a continuous value that directly reflects the current tool wear level.

## 4. Experimental Verification and Result Analysis

### 4.1. Experimental Setup

**Figure 6:**
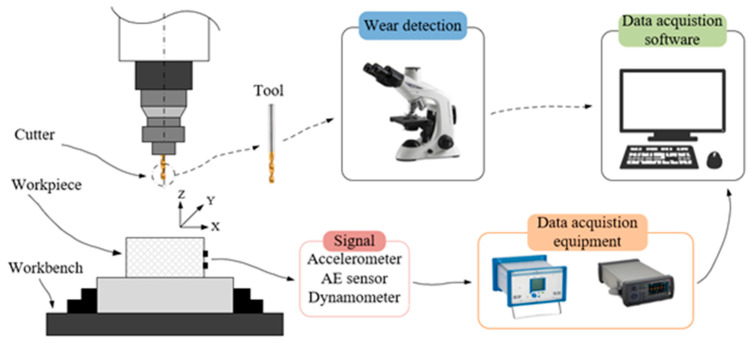
Experimental equipment diagram.

Experiments were conducted on the publicly available PHM Society 2010 dataset to evaluate the proposed method. The experimental procedure is illustrated in Figure 6, and Table 1 summarizes the equipment, sensors, and cutting parameters. The dataset includes six full life-cycle milling tests performed under identical conditions using climb milling with a milling length of 108 mm per pass. After each milling pass, the flank wear of the three cutting edges was measured using the microscope listed in Table 1. Three complete tool life cycles are provided for the ball-end milling cutters C1, C4, and C6, and each tool contains 315 passes with corresponding wear measurements. For each pass, seven sensor channels were recorded, including cutting-force components (Fx, Fy, Fz), vibration accelerations (Ax, Ay, Az), and the acoustic emission RMS signal (AE_rms). These multi-sensor signals capture wear-related information from complementary physical perspectives during machining. In this study, the average flank wear over the three cutting edges is used as the regression target for training and evaluation. This experimental setup provides a consistent benchmark for assessing both predictive accuracy and robustness.

### 4.2. Model Training

A complete training framework was implemented for the proposed CNN–ResNet–CBAM–BiGRU model to ensure accurate prediction and good generalization. A leave-one-tool-out protocol was applied to the three tools (C1, C4, C6), resulting in three folds; in each fold, two tools formed the development set and the remaining tool served as an independent test set. To avoid information leakage, the development set was split into training and validation subsets (80/20) in chronological order, and early stopping/model selection were based only on the validation loss. The held-out test tool was used only for final evaluation.

For model input, the seven-channel signals (Fx, Fy, Fz, Ax, Ay, Az, and AE_rms) were fused and reshaped into a two-dimensional feature matrix. Z-score normalization was applied to reduce distribution differences across tools. The model was trained for 500 epochs with a batch size of 100 and a learning rate of 0.001 using the Adam optimizer with L2 regularization. Mean squared error (MSE) was used as the optimization objective, and mean absolute error (MAE) was additionally reported for robustness. The random seed was fixed to 42 for reproducibility, and key architectural hyperparameters are summarized in Table 2.

### 4.3. Evaluation Indicators of the Experiment

To verify the feasibility and effectiveness of the proposed method, this study statistically quantifies the model performance using four performance evaluation metrics. These include: Mean Absolute Error (MAE), Root Mean Square Error (RMSE), and the Coefficient of Determination (R^2^). Smaller values of MAE and RMSE indicate stronger predictive ability and higher prediction accuracy of the model; while R^2^ ranges from 0 to 1, with larger values representing better predictive performance of the model. The calculation processes of each evaluation metric are shown in Equations (9)–(11).

(9) $$MAE=\frac{1}{N}\sum_{t=1}^{N}\left|\hat{X}(t)-X(t)\right|$$

(10) $$RMSE=\sqrt{\frac{1}{N}\sum_{t=1}^{N}(\hat{X}(t)-X(t){)}^{2}}$$

(11) $$R^{2}=1-\frac{\sum_{i=1}^{N}(\hat{X}(t)-X(t){)}^{2}}{\sum_{i=1}^{N}(X(t)-\bar{X}(t){)}^{2}}$$

### 4.4. Comparative Analysis

**Figure 7:**
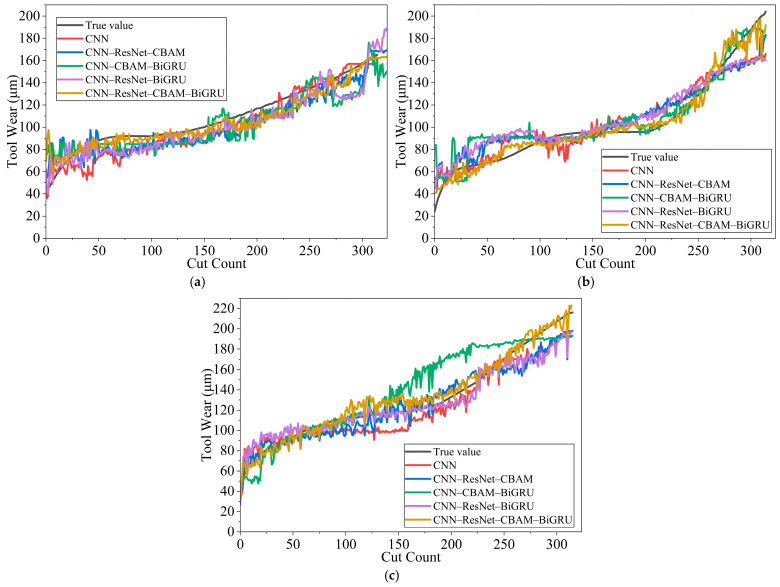
Comparison of prediction results for different models: (**a**) C1 Tool Different Model Prediction Results, (**b**) C4 Tool Different Model Prediction Results, (**c**) C6 Tool Different Model Prediction Results.

To evaluate the proposed CNN–ResNet–CBAM–BiGRU model, we compared it with four representative baselines (LSTM, BiLSTM, BiGRU, and CNN_LSTM), as listed in Table 3. LSTM uses a two-layer unidirectional architecture with 64 hidden units; BiLSTM employs bidirectional processing with an output dimension of 128; BiGRU reduces complexity while retaining temporal modeling capability; and CNN_LSTM combines a three-layer convolutional feature extractor with an LSTM temporal module. All models were trained under identical settings (500 epochs, batch size 100, learning rate 0.001, Adam optimizer) and used the same preprocessing procedures and evaluation metrics. The comparative results are shown in Figure 7.

In addition to predictive accuracy, computational cost and efficiency were reported to assess deployment feasibility. All experiments were implemented in Python 3.4 with PyTorch 1.13 on a CPU-only platform (Intel Core i5-8265U, 8 GB RAM). Table 4 summarizes the model size, training time, inference latency, and throughput under this environment. The proposed model contains 105,522 parameters (0.403 MB in FP32) and achieves an average inference latency of 0.00784 ± 0.00032 ms per sample (batch size 100), indicating that it is lightweight and suitable for fast online prediction.

The proposed method achieves the best overall results across all evaluation metrics. Relative to the strongest baseline (BiGRU), the average MAE decreases from 9.82 to 6.08 (38.1%), the average RMSE drops from 154.54 to 65.06 (57.9%), and the average R^2^ increases from 0.8687 to 0.9424 (8.5%). These improvements indicate that the proposed architecture alleviates limited feature extraction and vanishing-gradient issues. To enhance feature extraction, ResNet blocks and CBAM are jointly employed in the feature extraction module. On the C6 dataset, CBAM improves wear-related feature representation by adaptively reweighting responses in both channel and spatial dimensions while suppressing redundant information. As a result, the network focuses on wear-sensitive patterns in cutting-force features, providing more informative inputs for subsequent temporal modeling. To better handle long sequences while maintaining stable training, residual skip connections are combined with a BiGRU temporal module. This is evident when compared with CNN-LSTM: CNN-LSTM degrades on C6 (MAE = 17.35), whereas the proposed model remains stable across all three datasets (MAE coefficient of variation = 8.2%). Residual connections facilitate gradient backpropagation in the convolutional layers, and BiGRU captures long-range temporal dependencies, leading to more stable optimization. Overall, integrating CNN, ResNet, CBAM, and BiGRU yields complementary gains beyond those achieved by individual components. Across all datasets, the model achieves R^2^ > 0.91, demonstrating the effectiveness of the proposed hybrid architecture for tool wear prediction.

### 4.5. Ablation Experiment

**Figure 8:**
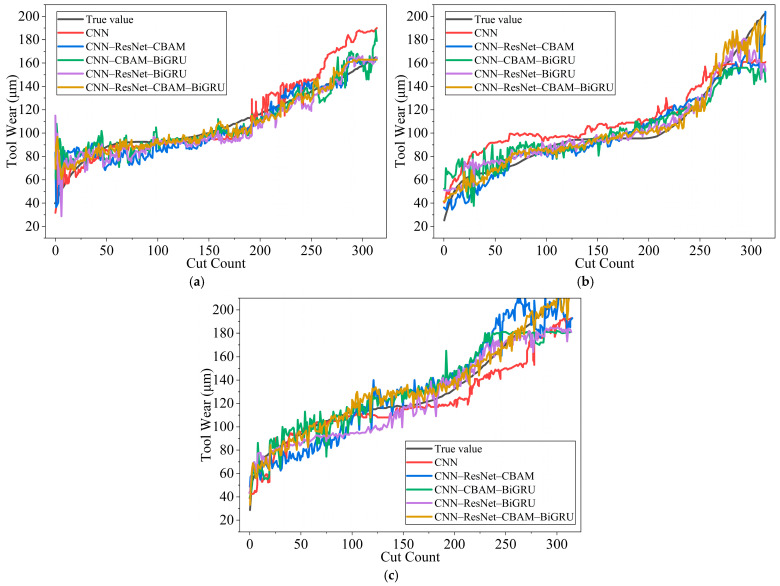
Comparison of ablation results for different model variants: (**a**) Comparison results of C1 tool ablation, (**b**) Comparison results of C4 tool ablation, (**c**) Comparison results of C6 tool ablation.

A systematic ablation study was performed to quantify the contribution of each component in the proposed CNN–ResNet–CBAM–BiGRU architecture. In each ablation variant, one module was removed while all other modules and training settings were kept identical to the full model. Five models were evaluated: (1) a baseline CNN, (2) CNN–ResNet–CBAM (without BiGRU), (3) CNN–ResNet–BiGRU (without CBAM), (4) CNN–CBAM–BiGRU (without ResNet), and (5) the full CNN–ResNet–CBAM–BiGRU model. Quantitative results are reported in Table 5, and the corresponding prediction curves are shown in Figure 8.

A systematic ablation study was conducted to quantify the contribution of each component in the proposed CNN–ResNet–CBAM–BiGRU model (Table 5). Overall, the full model achieves the best performance across all folds, reducing the average MAE from 11.59 (CNN) to 6.08 and improving the average R^2^ from 0.823 to 0.942, which confirms the effectiveness of the proposed multi-module design.

ResNet blocks are placed in the convolutional feature extraction stage before CBAM and BiGRU. This design enables deeper representation learning with stable gradient propagation via skip connections and provides richer intermediate feature maps for subsequent attention recalibration. Removing ResNet (CNN–CBAM–BiGRU) leads to a clear performance drop (average MAE: 6.08 → 9.79), demonstrating that residual learning is essential for optimization stability and generalization.

Temporal modeling is crucial for tool wear evolution. When BiGRU is removed (CNN–ResNet–CBAM), the average MAE increases to 10.21, and the degradation is most evident on the challenging C6 fold (MAE: 6.75 → 13.14), indicating that attention alone is insufficient to capture long-range wear dynamics under complex conditions. Moreover, CBAM yields additional gains when combined with BiGRU: compared with CNN–ResNet–BiGRU (average MAE = 8.14), the full model further reduces the average MAE to 6.08, showing that attention-based feature reweighting improves the quality of features fed into the temporal module.

In summary, the full CNN–ResNet–CBAM–BiGRU model demonstrates a clear synergy between residual learning (stable deep feature extraction), attention recalibration (feature selection), and bidirectional temporal modeling (wear evolution representation), resulting in the best overall accuracy and robustness.

### 4.6. Potential Industrial Impact and Limitations

In practical tool condition monitoring (TCM), multi-sensor signals can be collected during machining and a reliable wear indicator is required online. In addition to predictive accuracy, the proposed CNN-ResNet-CBAM-BiGRU model maintains a compact architecture (105,522 parameters, 0.403 MB in FP32) and achieves low inference latency on a CPU-only platform. Therefore, it can be integrated into edge devices or CNC controllers to provide tool-wear estimation after each cutting pass, which can support condition-based maintenance (CBM) and adaptive process control for early warning and parameter adjustment.

For industrial deployment, wear estimation becomes more valuable when it is linked to tool-replacement decisions. Compared with fixed-interval replacement, condition-based replacement can reduce premature tool changes and avoid late replacement that may cause excessive wear and scrap. Accordingly, reduced estimation error is expected to improve online replacement strategies (e.g., threshold- or cost-based decision rules). Recent studies also indicate that forecasting-oriented TCM, such as forecast-assisted multi-step wear/RUL prediction designed to avoid data leakage, can provide more actionable information for proactive replacement planning [35].

Several limitations remain and motivate future work. First, experiments were conducted on the PHM2010 dataset with three tools under constant cutting conditions; additional validation on different tool geometries/materials and varying cutting parameters is required to assess generalization. Second, the current implementation relies on seven sensor channels, which may not always be available in industrial environments; sensor selection/reduction and more robust fusion schemes are important for deployment. Third, tool wear is learned as a continuous regression target, which may be sensitive to measurement noise and may occasionally violate the physically monotonic degradation trend. To improve physical consistency, future work will model wear evolution using incremental values (Δ-wear) rather than absolute wear and enforce non-negativity/monotonicity (e.g., Δ-wear ≥ 0 followed by cumulative summation) to reduce fluctuations and better reflect the degradation process. In addition, the framework will be extended from wear monitoring to wear forecasting (multi-step ahead prediction or remaining useful life estimation) to further enhance its industrial applicability.

## 5. Conclusions

This paper presents a hybrid tool-wear prediction method based on a 34-dimensional multi-domain feature system and a CNN–ResNet–CBAM–BiGRU architecture, and the following conclusions are drawn:

A 34-dimensional feature set (time, frequency, and time–frequency domains) is constructed. CNN extracts spatial features, ResNet stabilizes deep optimization, CBAM assigns adaptive attention weights, and BiGRU models bidirectional temporal dependencies, thereby improving feature learning and temporal modeling.

On PHM2010 (leave-one-tool-out, three tools), the proposed method achieves MAE = 6.08, RMSE = 65.06, and R^2^ = 0.9424, improving over the best baseline (BiGRU) by 38.1%, 57.9%, and 8.5%, respectively. Ablation results show that BiGRU brings the main gain (29.8% MAE reduction vs. CNN), CBAM adds 11.9%, and the full model yields 47.5% overall improvement, indicating a synergistic effect.

Future work will focus on sensor reduction and robust fusion (given the current seven-channel dependence), broader validation beyond PHM2010, and online adaptation via cross-dataset transfer learning and incremental updating.

## Figures and Tables

**Table 1:** Types of Experimental Equipment and Cutting Conditions.

Experimental Equipment	Product Model	Cutting Parameters	Value
Machine tool	Roders Tech RFM760 (Röders GmbH, Soltau, Germany)	Spindle speed (r/min)	10,400
Cutting tools	Ball-end carbide milling cutter	Feed speed (mm/min)	1555
force gauge	Kistler 9265B (Kistler Instrumente AG, Winterthur, Switzerland)	Axial cutting depth (mm)	0.2
Vibration sensor	Kistler 8636C (Kistler Instrumente AG, Winterthur, Switzerland)	Radial cutting depth (mm)	0.125
Charge amplifier	Kistler 5019A (Kistler Instrumente AG, Winterthur, Switzerland)	Feed per cutting (mm)	0.001
Data acquisition card	NI DAQ (National Instruments, Austin, TX, USA)	Sample frequency (kHz)	50
microscope	LEICA MZ12 (Leica Microsystems GmbH, Wetzlar, Germany)	Sample frequency (kHz)	Dry cutting

**Table 2:** Key architectural hyperparameters of the CNN–ResNet–CBAM–BiGRU model.

Network Layer	Parameters
ResidualBlock 1	Conv2d = 3 × 3, stride = 1, padding = 1
CBAM block	spatial kernel size = 7; reduction ratio = 16
ResidualBlock 2	Conv2d = 3 × 3, stride = 1, padding = 1
BiGRU layer	input size = 7; hidden size = 64; num layers = 2; dropout = 0.1

**Table 3:** Evaluation indicators of each comparison model.

Method	Evaluation Indicators			
	Test Data	MAE	RMSE	*R* ^2^
LSTM	C1	8.37	11.36	0.8268
	C4	10.87	13.47	0.8738
	C6	10.68	12.64	0.9006
BILSTM	C1	8.73	10.40	0.8550
	C4	10.84	13.60	0.8713
	C6	9.68	11.65	0.9155
CNN-LSTM	C1	8.63	11.32	0.8279
	C4	10.03	12.60	0.8896
	C6	17.35	20.32	0.7429
BIGRU	C1	9.42	11.30	0.8318
	C4	11.80	14.47	0.8543
	C6	8.23	11.34	0.9198
CNN-ResNet-CBAM-BIGRU	C1	5.70	8.05	0.9132
	C4	5.76	7.97	0.9558
	C6	6.75	8.18	0.9583

**Table 4:** Runtime and model size.

Fold (Test Tool)	Total Train Time (s)	Avg Epoch Train (s)	Avg Epoch Test (s)	Model Size (MB)	Inference (ms/Sample)	Throughput (Samples/s)
C1	18.152	0.02777	0.00705	0.403	0.00821	121,802
C4	19.782	0.03024	0.00745	0.403	0.00771	129,627
C6	16.967	0.02611	0.00665	0.403	0.00760	131,561
Mean ± Std	18.30 ± 1.41	0.02804 ± 0.00208	0.00705 ± 0.00040	0.403	0.00784 ± 0.00032	127,663 ± 5167

**Table 5:** Evaluation indicators of each ablation model.

Method	Evaluation Indicators			
	Test Data	MAE	RMSE	R^2^
CNN	C1	14.46	16.52	0.8103
	C4	10.05	13.19	0.7668
	C6	10.26	13.16	0.8921
CNN-ResNet-CBAM	C1	8.01	10.05	0.8647
	C4	9.49	12.03	0.9013
	C6	13.14	16.15	0.8377
CNN-CBAM-BiGRU	C1	11.97	14.80	0.8635
	C4	9.08	12.69	0.8880
	C6	8.32	11.86	0.8794
CNN-ResNet-BiGRU	C1	7.03	10.82	0.8431
	C4	7.12	11.27	0.9117
	C6	10.27	13.07	0.8937
CNN-ResNet-CBAM-BiGRU	C1	5.70	8.05	0.9132
	C4	5.78	7.97	0.9558
	C6	6.75	8.18	0.9583

## Data Availability

The data used in this study are publicly available from the PHM Society 2010 PHM Society Conference Data Challenge (CNC milling cutter wear dataset) at https://phmsociety.org/phm_competition/2010-phm-society-conference-data-challenge/ (6 November 2025). No new data were created in this study.
